# Synchronous Immobilization of Arsenic and Cadmium in Agricultural Soils by Sepiolite-Supported Nanoscale Zero-Valent Iron

**DOI:** 10.3390/toxics14040301

**Published:** 2026-03-31

**Authors:** Kuiru Li, Tieguang He, Yan Wang, Xinru Wang, Donghuan Lei, Lijuan Li

**Affiliations:** 1 Guangxi Key Laboratory of Agroenvironment and Agroproduct Safety, College of Agriculture, Guangxi University, Nanning 530004, China; yllkr1663229302@163.com (K.L.); wangxinru2025@163.com (X.W.); 15307860649@163.com (D.L.); 2 Agricultural Resources and Environmental Research Institute, Guangxi Academy of Agricultural Sciences/Guangxi Key Laboratory of Arable Land Conservation, Nanning 530007, China; tghe118@163.com; 3 College of Environmental Science and Engineering, China West Normal University, Nanchong 637009, China; zty2570699599@163.com

**Keywords:** sepiolite, nano zero-valent iron, arsenic–cadmium cocontamination, immobilization mechanisms, soil remediation

## Abstract

The cocontamination of arsenic (As) and cadmium (Cd) in agricultural soils poses severe risks to ecosystem stability and food safety because of their high toxicity, mobility, and bioaccumulation potential. However, single amendments often exhibit selective immobilization, which limits their effectiveness for As–Cd-cocontaminated soils. In this study, a sepiolite-supported nanoscale zero-valent iron composite (S-nZVI) was synthesized via liquid-phase reduction, and its remediation performance and mechanisms under different moisture conditions were evaluated. The characterization results confirmed that the nZVI nanoparticles were uniformly dispersed and anchored onto the sepiolite matrix, thus mitigating aggregation and oxidative passivation while increasing surface reactivity. Soil incubation experiments demonstrated that S-nZVI reduced the bioavailability of As and Cd and promoted their transformation from labile to stable fractions under both 50% and 120% water holding capacity (WHC). Under flooded conditions (120% WHC), 0.5% S-nZVI reduced the bioavailable Cd and As concentrations by 52.3–58.7% and 67.4%, respectively, after 120 days. Mechanistically, immobilization was governed by a synergistic “adsorption–reduction–coprecipitation” pathway coupled with pH–Eh regulation. Rice pot experiments further validated the effectiveness of S-nZVI, with the grain As and Cd concentrations reduced by 73.3% and 52.3%, respectively, without impairing plant growth. Overall, S-nZVI provides an efficient strategy for simultaneous immobilization of As and Cd in As–Cd-cocontaminated soils and supports the safe use of polluted agricultural lands.

## 1. Introduction

With the rapid expansion of industrialization and intensive agricultural practices, the problem of heavy metal pollution in agricultural soil has become increasingly prominent. Composite pollution by arsenic (As) and cadmium (Cd) has become a key environmental issue that restricts the sustainable development of agriculture and threatens food security because of their high toxicity, strong mobility, and significant bioaccumulation characteristics [1]. As and Cd are both classified as Group 1 carcinogens by the International Agency for Research on Cancer (IARC) and can accumulate through the soil-crop-human food chain in agricultural ecosystems. These elements frequently cooccur in soils because of their common geochemical associations and coemissions from anthropogenic activities such as mining, smelting, and the long-term application of phosphate fertilizers that contain trace metal impurities [2,3]. Especially in rice planting systems, changes in the redox environment induced by flooding management methods exacerbate their environmental risks [4,5,6,7]. Therefore, the development of efficient, stable, and environmentally friendly remediation technologies for As and Cd complex-contaminated agricultural soil has not only significant theoretical and practical implications but also outstanding practical significance.

In response to the problem of As and Cd pollution, scholars in China and other countries have carried out many studies and proposed a variety of in situ passivation and stabilization technical paths. Previous studies have shown that clay minerals, iron-based materials, and their modified materials can reduce the bioavailability of heavy metals through processes such as adsorption, complexation, precipitation, and redox [8,9,10]. However, in the As and Cd composite pollution system, owing to the differences in chemical form, charge properties, and migration and transformation behaviors between the two elements, a single repair material often exhibits obvious selective passivation characteristics, which makes achieving simultaneous stabilization difficult. Clay minerals have strong ion-exchange and physical adsorption capabilities for Cd, but their ability to fix As in anionic form is limited [11,12,13]; although iron-based materials can effectively regulate the valence state of As and promote its fixation, their long-term stabilizing effect on Cd may weaken because of oxidative deactivation or changes in environmental conditions [14,15,16]. For example, the fixation efficiency of hydroxyapatite for Cd can reach 85%, but it promotes the activation and release of As, and the application of lime can alter the pH. Although the application of lime can promote the precipitation of Cd, it increases the dissolution and migration ability of As [17]. Additionally, flooded reducing conditions are beneficial for the fixation of Cd, but these conditions trigger the conversion of As(V) into highly toxic As(III) [18]. This antagonistic remediation behavior represents a major limitation for practical engineering applications. Additionally, in plants, As can promote plant absorption of Cd by regulating the expression of iron transporters, whereas Cd inhibits As transport by competing for ionophores [19]. This interaction mechanism leads to an increase in combined toxicity, which is manifested as synergistic oxidative stress damage and functional disturbance of soil microbial communities. Addressing such trade-off effects has become a critical challenge in the remediation of cocontaminated soils.

In this context, composite passivation materials have attracted widespread attention because of their potential synergistic mechanisms. As a layered-chain silicate mineral, sepiolite has the advantages of a large specific surface area, a well-developed pore structure, and abundant surface and active sites, and has good adsorption potential for the remediation of Cd-contaminated soil [20]. Nanoscale zero-valent iron is outstanding for the fixation and valence control of As because of its strong reducing ability and iron oxide generation ability [21,22]. However, nanoscale zero-valent iron is prone to agglomeration and rapid oxidation in environmental media, which severely restricts its reactivity and long-term stability. Previous studies have shown that loading nanoscale zero-valent iron onto porous mineral carriers is an effective way to improve its dispersion and stability and increase repair efficiency [23,24,25]. This finding provides an important strategy for the construction of efficient As and Cd synergistic repair materials. Denny et al. prepared sugarcane bagasse biochar loaded with nanoscale zero-valent iron for the adsorption of the heavy metal chromium in water and reported that the modified material could effectively remove Cr(VI) at a ratio of 3:1 [26]. After in-depth research, Ainiwaer et al. revealed the efficient adsorption mechanism of Cd by sepiolite-loaded nanoscale zero-valent iron (S-nZVI), thus confirming that S-nZVI is a material with excellent adsorption performance for Cd and has broad application potential in the field of water and soil remediation for only mild Cd pollution [27].

Building upon the above foundation, in this study, S-nZVI is developed, and its performance in the simultaneous immobilization of As and Cd in complex soil systems—a critical challenge distinct from simplified aqueous environments—is evaluated. By integrating the adsorption and ion-exchange properties of sepiolite with the reduction and coprecipitation capabilities of nano-zero-valent iron, the regulatory effects of the material on soil physicochemical properties, metal availability, and fractionation are systematically assessed under varying moisture conditions. Crucially, this study validates the effects of the material on crop growth and heavy metal accumulation in a rice pot system, thereby directly linking soil remediation to agricultural safety. This work not only offers a simplified and efficient approach for the simultaneous remediation of As- and Cd-cocontaminated agricultural soils but also provides practical insights into the management of heavy metal uptake in crops, thus advancing the development of synergistic remediation strategies for complex pollution scenarios.

## 2. Materials and Methods

**Chemical materials and test samples.** All the chemicals were of analytical grade and were used without further purification. The natural sepiolite (SEP) used in this study was purchased from Sigma-Aldrich (Shanghai) Trading Co., Ltd., Shanghai, China and the zero-valent iron (nZVI) was purchased from Zhongke Leiming (Beijing) Co., Ltd., Beijing, China. NaBH_4_ and FeCl_3_·6H_2_O were purchased from Chengdu Jinshan Chemical Reagent Co., Ltd., Chengdu, China. CaCl_2_·2H_2_O, (NH_4_)_2_SO_4_, CH_4_N_4_S, NH_4_H_2_PO_4_, (NH_4_)_2_C_2_O_4_, C_6_H_8_O_6_, and NH_2_OH·HCl were purchased from the China National Pharmaceutical Collection, Shanghai, China. CH_3_COONH_4_ and C_6_H_15_NO_3_ was obtained from Tianjin Fuchen Chemical Reagent Co., Ltd., Tianjin, China. C_14_H_23_N_3_O_10_ was obtained from Tianjin Comio Chemical Reagent Co., Ltd., Tianjin, China. C_2_H_5_OH, CH_3_COOH, H_2_O_2,_ HCl, and HNO_3_ were purchased from Kelong Chemical Co., Ltd., Chengdu, China. The experimental water was deionized water.

The test soil was collected from an agricultural area surrounding a mining area in Liuyang city, Hunan Province (latitude: 28°22′, longitude: approximately 113°45′). The basic properties and heavy metal contents of the tested soil are presented in Table 1. The soil from the top 0–20 cm of the cultivated layer was naturally air-dried, impurities were removed, and the soil was passed through a 2 mm sieve and mixed until use. The soil tested was acidic, and the As and Cd contents significantly exceeded the limits of the “Soil Environmental Quality—Agricultural Land Soil Pollution Risk Management and Control Standards” (GB 15618-2018) [28]; thus, the soil was a composite-contaminated soil of As and Cd. The rice variety tested was wild-type Nipponbare (*Oryza sativa* L. cv. Nipponbare).

**Synthesis of S-nZVI.** S-nZVI was prepared by an improved liquid-phase reduction method. First, natural sepiolite was placed in 2 mol L^−1^ of HCl solution and magnetically stirred at 80 °C for 8 h for acid pretreatment to remove impurities and increase the specific surface area. Afterward, the samples were repeatedly washed with deionized water until no Cl^−^ remained, after which they were dried and set aside.

**Figure 1:**
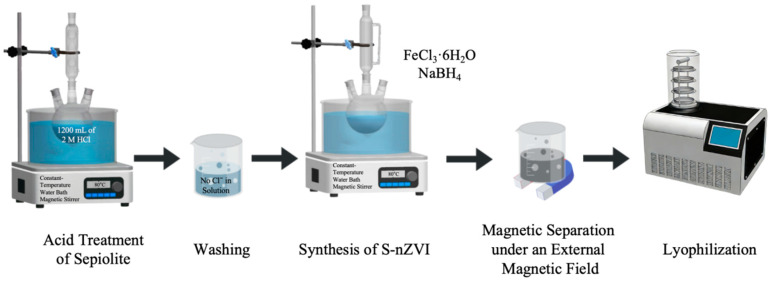
Synthesis process for S-nZVI.

The pretreated sepiolite was dispersed in the FeCl_3_·6H_2_O solution. After thorough stirring and ultrasonic dispersion, the NaBH_4_ solution was slowly added dropwise to perform the reduction reaction. Zero-valent iron was generated under continuous stirring conditions and loaded onto the surface of sepiolite in situ. After the reaction was completed, the solid product was separated by applying an external magnetic field, sequentially washed with deionized water to remove residual ions, freeze-dried, and sealed for later use. The reaction process can be expressed in Figure 1:

$$4Fe^{3+}+3BH_{4}^{-}+9H_{2}O\to 4Fe^{0}+3H_{2}BO_{3}^{-}+6H_{2}+12H^{+}$$

**Characterization of S-nZVI**. In this study, the surface morphology and microstructure of the synthesized material were observed. At 5 kV, the morphological characteristics and particle size of the adsorbed material surface were measured by scanning electron microscopy (SEM, HORIBA EMAX mics2). The specific surface area of the synthesized S-nZVI sample was determined through nitrogen adsorption and desorption curve testing. The Brunauer–Emmett–Teller (BET) surface area was determined using a Micromeritics-ASAP-2460 automatic analyzer. In addition, the pore size of the sample was analyzed by the Barrett–Joyner–Halenda (BJH) calculation method. The crystal structure of the synthetic material was measured by X-ray diffraction (XRD) using a German Bruker D8-ADVANCE X-ray diffractometer. The tube current was 40 mA, the tube voltage was 40 kV, the Cu target wavelength was 1.5406 Å, and the Co target wavelength was 1.79026 Å. The chemical valence state of the surface of the synthetic material was determined by X-ray photoelectron spectroscopy (XPS). The instrument used was an American Thermo ESCALAB 250Xi microprobe. The relevant parameters were monochromatic Al Kα (hν = 1486.6 eV), a power of 150 W, a 650 μm beam spot, a voltage of 14.8 kV, and a current of 1.6 A. The charge was corrected using contaminated carbon C 1s = 284.8 eV. The full-spectrum pass energy was 100 eV, with a step size of 1 eV, and the narrow-spectrum pass energy was 20 eV, with a step size of 0.1 eV.

**Soil incubation experiment.** A controlled soil incubation experiment was conducted to study the effects of various passivation materials on the physical and chemical properties of soil. The experiments included a blank control group (Control Check, CK) and three passivation treatment groups: sepiolite (SEP), nano-zero-valent iron (nZVI), and S-nZVI. Each treatment group was treated with two concentrations (1 g/kg and 5 g/kg). The specific experimental design was as follows: One kilogram of air-dried soil (<2 mm) was thoroughly mixed with the designated amendment. In the control group, passivating agents were not added. In the experimental group, SEP, nZVI, or S-nZVI was added according to the designed concentration, and the compound was mixed thoroughly. Either no passivator (CK), 0.1% SEP (SEP-1), 0.5% SEP (SEP-5), 0.1% nZVI (nZVI-1), 0.5% nZVI (nZVI-5), 0.1% S-nZVI (S-nZVI-1), or 0.5% S-nZVI (S-nZVI-5) was added. The maximum field water holding capacity (WHC) of the soil was used as the benchmark for water regulation, and two water treatments were established: 50% WHC (drought conditions) and 120% WHC (flooded conditions). The maximum field water holding capacity (WHC) of the soil was determined using the gravimetric method described by Cassel and Nielsen [29]. Deionized water was regularly replenished through the weighing method to maintain the set water content. All the treated samples were placed in a constant-temperature incubator (25 ± 1 °C) for a 120-day culture experiment, and each group of treatments was repeated three times. A total of 7 sampling time points were established during the culture cycle (7, 15, 30, 45, 60, 90, and 120 days). Soil samples were collected at each time point and freeze-dried, ground, and sieved before use.

**Figure 2:**
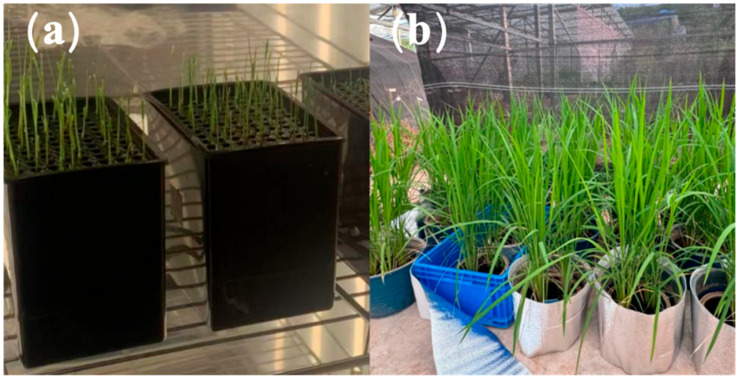
Rice pot experiment: (**a**) Rice seedling cultivation and (**b**) rice planting.

**Rice pot experiment.** Rice seeds were surface-sterilized with 2% H_2_O_2_ solution and soaked at 40 °C for 24 h. After sterilization, the seeds were thoroughly rinsed with deionized water to remove residual chemicals and subsequently transferred to Petri dishes that contained preheated deionized water (40 °C) for germination under constant-temperature conditions. Once the embryos elongated to approximately 0.5 cm, the germinated seeds were evenly sown in 96-well seedling trays and incubated in the dark at 25 °C to promote root development. After emergence, the seedlings were transferred to a light incubator under a photoperiod of 12 h light/12 h dark at 24–30 °C and cultivated with half-strength Hoagland nutrient solution. High humidity was maintained during the initial stage of seedling growth and was gradually adjusted to normal growth conditions after three days. After 3–4 weeks of cultivation (the three-leaf stage), uniformly growing seedlings were selected for pot experiments. The rice seedling cultivation process is illustrated in Figure 2a.

The results of the rice pot experiments are shown in Figure 2b. Four groups were established: a control (CK) group and three groups treated with the amendments SEP, nZVI, and S-nZVI. Each amendment was applied at two doses (1 g kg^−1^ soil and 5 g kg^−1^ soil).

Approximately 3 kg of air-dried soil that had passed through a 2 mm sieve was placed into polyethylene pots (with a height of 17.6 cm, an upper diameter of 23 cm, a bottom diameter of 15.7 cm). The corresponding amendments and fertilizers were thoroughly mixed with the soil prior to transplantation. Each pot received chemical fertilizers at the following rates: 0.20 g N [CO(NH_2_)_2_], 0.12 g P (KH_2_PO_4_), and 0.26 g K (K_2_SO_4_). After mixing, deionized water was added to maintain flooded conditions approximately 5 cm above the soil surface, and the soils were equilibrated for one week before transplanting.

Uniform rice seedlings were then transplanted into each plot. During the cultivation period, the water level was maintained at approximately 2 cm above the soil surface using deionized water. Plant height was measured at three key growth stages of rice (tillering, heading, and maturity). After harvest, rice tissues were collected for subsequent analysis.

**Data collection.** The amount of available Cd in the soil was measured using the DTPA extraction method, and the amount of available As was measured using the modified DTPA-atomic fluorescence method. After centrifugation and filtration, the extract was measured using ICP-OES (Agilent 5110) and an atomic fluorescence spectrometer (AFS; Ji Tian, China), respectively. The improved Wenzel continuous extraction method was used to determine the forms of As in the soil, which were divided into nonspecific adsorption states, specific adsorption states, iron-aluminum oxide combined states, and residual states. The existing forms of Cd were determined via the four-step BCR continuous extraction method and were divided into weakly acidic dissolved states, reduced states, oxidized states, and residual states. After the rice stems and grains were dried and digested, ICP-OES was used to determine the As and Cd contents. The ability of rice to accumulate heavy metals was evaluated on the basis of the enrichment coefficient, and its calculation formula is as follows:

The above-ground enrichment factor (shoot enrichment factor, *EF_shoot_*) reflects the ability of stems to enrich soil heavy metals:

$${EF}_{shoot}=\frac{C_{shoot}}{C_{rhi}}$$

where *C_shoot_* represents the heavy metal content in the shoot, and *C_rhi_* represents the heavy metal content in the rhizosphere soil.

The grain enrichment factor (*EF_grain_*) directly reflects the degree of enrichment of soil heavy metals by grains:

$${EF}_{grain}=\frac{C_{grain}}{C_{rhi}}$$

where *C_grain_* represents the heavy metal content in the shoot, and *C_rhi_* represents the heavy metal content in the rhizosphere soil.

In accordance with the Chinese national food safety standards for food contaminants (GB 2762-2022) [30], the maximum permissible concentrations of Cd (Cd) and inorganic As (As) in rice grain are both 0.2 mg kg^−1^. These regulatory thresholds provide important benchmarks for evaluating the safety of rice produced from contaminated soils.

**Data analysis.** Statistical analysis was performed using Microsoft Excel and IBM SPSS Statistics 27 software. One-way analysis of variance was used to analyze significant differences between the control and treatment groups, with *p* < 0.05 indicating a significant difference. Origin 2022 was used for mapping.

## 3. Results

### 3.1. Material Characterization

SEM revealed that the SEPs gathered together as flakes with uneven surfaces (Figure S1a). The surface of S-nZVI was rougher, and the n-ZVI particles were evenly dispersed on the SEP surface, with no obvious agglomeration observed (Figure S1b). In addition, some n-ZVI particles entered the pores of sepiolite, which reduced its contact area with the external environment. Energy dispersive X-ray (EDX) results also revealed a high Fe peak for S-nZVI, which indicated that nZVI was loaded well into the SEP (Figure S1c).

The N_2_ adsorption-desorption isotherm (Figure S2a–c) shows that S-nZVI has a type IV isotherm. An H3-type hysteresis ring (P/P_0_ > 0.5) was observed in S-nZVI, which indicated the presence of a mesoporous structure (2–50 nm) in the material. The pore size distribution curve shows that the average pore size of S-nZVI was approximately 7.89 nm, thus indicating that S-nZVI has a nanosized pore structure that is suitable for applications that involve contaminant adsorption. The BET test results revealed that the specific surface area of S-nZVI was 120 m^2^/g and that the specific surface area of the untreated sepiolite powder was 87 m^2^/g, which indicated that the specific surface area of the material after composite modification increased significantly. In addition, from the perspective of the adsorption capacity (Figure S2c), a comparison of SEP with S-nZVI revealed that the adsorption capacity after modification significantly increased, which indicated that the modified material inherited the pore structure of the original material and had more abundant mesopores.

The crystal structures of the SEP, nZVI, and S-nZVI composites were characterized using X-ray diffraction (XRD) (Figure S3). The typical diffraction peaks observed for SEP at 2*θ* = 17.2°, 20.6°, 26.9, 27.8, 30.9°, 36.8°, 39.3°, and 41.2° were assigned to the characteristic planes of sepiolite (PDF#13-0595), namely, (150), (060), (080), (261), (331), (281), and (210), respectively. Among the series of characteristic diffraction peaks in the range of 2θ = 21–31°, the strong peak at 2θ ≈ 26–31° can be attributed to the typical diffraction signal of the SiO_2_ crystal phase in sepiolite, whereas the diffraction peak at 2θ ≈ 29.3° is highly consistent with the crystal structure of CaCO_3_, thus indicating that there may have been partial substitution of Ca^2+^ in the mineral structure of sepiolite. In the S-nZVI composite, these main characteristic peaks of sepiolite were still detectable, which indicated that the sepiolite framework was largely preserved after composite synthesis. However, compared with those of pristine SEP, some peaks of S-nZVI were less intense and/or slightly broader, which suggested that nZVI loading affected the crystallographic response of the carrier. In addition, only a weak characteristic peak of zero-valent iron (Fe^0^) appeared at 2θ ≈ 44.8° (PDF#06-0696). In addition, a weak characteristic broad peak of zero-valent iron (Fe) appeared at 20 = 44.8°, which is in accordance with the XRD result reported by Antony et al. (2022) [31] and Taha et al. (2014) [32]. This peak can be attributed to the (110) crystal plane diffraction of Fe^0^, thus indicating that sepiolite can effectively load and stabilize zero-valent iron during the synthesis process.

X-ray photoelectron spectroscopy (XPS) was used to analyze the chemical valence state of iron on the surface of the S-nZVI composite material (Figure S4a–c). The XPS results reveal that C 1s, O 1s, and Fe 2p were present in the sample, and their binding energies were approximately 284.8 eV, 530.0 eV, and 710.0 eV, respectively. The high-resolution C 1s spectrum can be divided into three chemical bonds: C–C (284.8 eV), C=O (287.8 eV), and –COOH (≈289.0 eV). The O 1s spectrum analysis revealed that oxygen existed mainly in the form of hydroxyl oxygen (531.5 eV) and adsorbed water (533.0 eV). The Fe 2p spectrum exhibited Fe 2p_3_/_2_ and Fe 2p_1_/_2_ characteristic peaks at ~706–710 eV and ~723–730 eV, respectively. The peak at 706 eV indicated that Fe^0^ was successfully synthesized onto the SEP. Further peak fitting results revealed that Fe^2+^ and Fe^3+^ coexisted on the material surface. The Fe^3+^ 2p_3_/_2_ peak at 710.5–711.5 eV was observed, and an obvious satellite peak was observed at 719 eV, which indicated the presence of Fe^3+^ species in the sample. The Fe^2+^ 2p_3_/_2_ peak and Fe^2+^ 2p_1_/_2_ peak were also identified at ~710.0 eV and ~722.0 eV, respectively. The above results indicate that iron existed mainly in the mixed valence state in the composite material. Some potential iron oxides, such as hematite, geothite, or ferrihydrite, may have formed to increase the adsorption ability of S-nZVI.

### 3.2. Effects on Soil pH and Redox Potential

Under different moisture conditions, the soil pH in each treatment group changed to varying degrees with culture time (Figure S5). Overall, the increase in soil pH under the 120% WHC condition was greater than that under the 50% WHC condition. Under the 50% WHC condition, the soil pH of the SEP treatment group was greater than that of the control group throughout the entire culture process and remained relatively stable during the later stages of culture. In the nZVI treatment group, the soil pH rapidly increased during the early stages of culture but then tended to decrease over time. In contrast, the S-nZVI treatment group showed a continuous increase in pH during the cultivation process, and the fluctuation range was small. There were differences in the degree of increase in soil pH under different amounts of addition. The treatment group with higher amounts of added compounds had an overall higher pH value. Under the 120% WHC condition, each additive treatment increased the soil pH to varying degrees, with the S-nZVI treatment group maintaining a relatively high pH level during the later stages of culture.

Under different moisture conditions and treatments, the soil Eh clearly changed with the culture time, and its change trend was opposite to that of the soil pH (Figure S5). Under the 50% WHC condition, the soil Eh in the nZVI treatment group rapidly decreased during the early stages of culture and remained at a low level, with certain fluctuations throughout the culture period. The soil Eh in the SEP treatment group continued to decrease compared with that in the control group. The S-nZVI treatment group also showed a decrease in soil Eh, but the fluctuation amplitude was relatively small. Under the condition of 120% WHC, the soil Eh in each treatment group decreased, among which the greatest decrease occurred in the initial stage in the nZVI treatment group, after which it rebounded to a certain extent. In contrast, the changes in Eh in the SEP and S-nZVI treatment groups were relatively small and remained relatively stable throughout the culture process.

### 3.3. Effects on the Availability of Cd and As in the Soil

**Figure 3:**
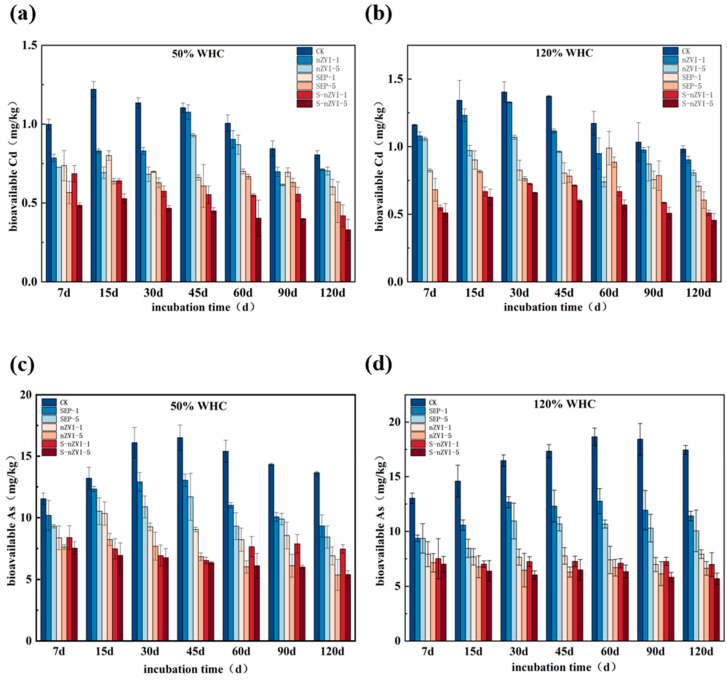
Dynamics of bioavailable (**a**,**b**) Cd and (**c**,**d**) As in soils under 50% and 120% WHC treatments during soil incubation experiments on different days.

The results of the soil culture experiments revealed that under different moisture conditions and different treatments, the contents of available Cd and available As in the soil decreased to varying degrees compared with those in the CK group (Figure 3). The content of available Cd in the CK group increased slightly at the beginning of culture, but then stabilized and remained at a high level overall. Compared with the CK group, the nZVI treatment group had a certain reduction in available Cd, and its available Cd content was reduced by 8% and 17.9%. The reduction in the SEP treatment group was more obvious, and compared with that in the CK group, the available Cd content in the SEP treatment group was reduced by 28% and 38.4%, respectively. In the S-nZVI treatment group, the decrease in the available Cd was the most significant. S-nZVI-5 always maintained the lowest available Cd content throughout the entire culture process, which was **52.3–58.7%** lower than that in the CK group, thus indicating better passivation stability **(*p* < 0.05)**. Under 50% WHC, each passivator treatment reduced the available As content in the soil. Compared with that in the CK group, the available As content in the SEP-5 treatment group decreased from 13.66 mg/kg to 8.43 mg/kg after 120 days of culture, whereas the available As content in the nZVI-5 treatment group decreased by 61%. Compared with that in the CK group, the As passivation effect in the S-nZVI treatment group was significant, and compared with that in the control group, the effective As content in the S-nZVI treatment group was reduced by **60.43% (*p* < 0.05)**. Under the 120% WHC flooding condition, the available As content in the CK group was generally higher than that under the 50% WHC condition and continued to increase in the early stages of culture and then decreased in the later stages, but remained at a high level. In contrast, compared with those in the CK group, the available As contents in the nZVI and S-nZVI treatment groups remained low throughout the entire culture process, with those in the nZVI-5 and S-nZVI-5 groups decreasing by 62.00% and 67.35%, respectively. Overall, under different moisture conditions, the effective contents of Cd and As decreased in the S-nZVI treatment groups. 

**Figure 4:**
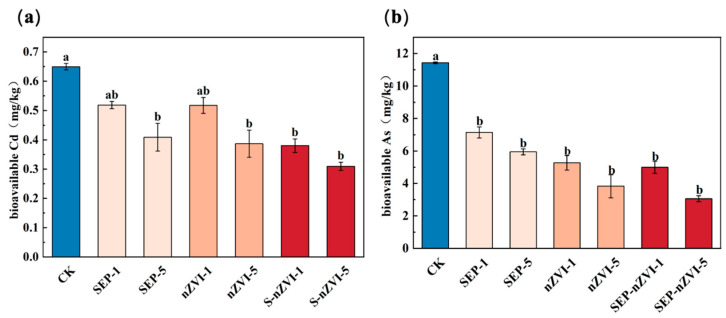
Effects of different passivation treatments on bioavailable Cd and As in rhizosphere soil during potted rice experiments. ((**a**): Cd; (**b**): As). (a, b indicate significant differences between groups at *p* < 0.05; ab indicates no significant difference from groups a and b).

The potted rice experimental results revealed that after the addition of different passivators, the available Cd and available As contents in the rice rhizosphere soil tended to decrease to varying degrees (Figure 4). After 0.5% SEP was added, the available Cd content in the rhizosphere soil decreased to 0.40 mg/kg (*p* < 0.05), which was 24.7% lower than that in the CK group. Compared with the control group, nZVI-5 treatment reduced the content of available Cd to a very low level. In contrast, the effect for the S-nZVI treatment group was more obvious. Both S-nZVI-1 and S-nZVI-5 showed extremely significant differences, with the effective Cd content reduced by **41.46%** and **52.28%,** respectively **(*p* < 0.05)**, thus indicating that S-nZVI has a strong passivation ability for Cd. The effects of different passivating agent treatments on the available As content in the rice rhizosphere soil also significantly differed. Compared with the CK treatment, the nZVI-1 treatment reduced the available As content to 5.28 mg/kg, which was a decrease of 53.82%, and the high-concentration nZVI-5 treatment further reduced it to 3.82 mg/kg, which was a decrease of 66.53%. Compared with the CK treatment alone, the SEP treatment alone resulted in a relatively small reduction in available As, and the available As content was reduced by 37.48% and 47.92%, respectively. The S-nZVI treatment group showed a more significant As passivation effect. After treatment with S-nZVI-1 and S-nZVI-5, the effective As content decreased to 5.00 mg/kg and 3.00 mg/kg, respectively, which were **56.21%** and **73.27%** lower than that in the CK group **(*p* < 0.05)**. The S-nZVI-5 treatment group reached the lowest value among all the treatment groups.

### 3.4. Effects on the Fractions of Cd and As in the Soil

The BCR continuous extraction results revealed that the distributions of Cd and As forms in the soil changed significantly under different moisture conditions and treatments (Figures S6–S9). Under 50% WHC conditions, Cd in the CK group was mainly in the weakly acid-soluble state (F1) and the reducible state (F2), with the proportion of F1 maintained at a high level. Compared with the CK treatment, both the SEP treatment and the nZVI treatment reduced the proportion of F1, which was accompanied by an increase in the proportion of residual Cd (F4). Among them, the S-nZVI treatment had the most significant effect on Cd transformation. Under the S-nZVI-1 and S-nZVI-5 treatments, the proportion of F4 increased to 24.62% and 28.10%, respectively, whereas the proportion of F1 decreased by 12.38% and 14.51%, respectively, compared with those in the CK group. Under the condition of 120% WHC, F1 was still the main form of Cd in the soil, but as the culture time increased, each passivator treatment promoted the conversion of F1 to F4, and the final proportion of F4 under high-moisture conditions was greater than that at 50% WHC. In terms of As, under 50% WHC conditions, the proportions of nonspecifically adsorbed As (F1) and specifically adsorbed As (F2) were greater in the CK group. After passivator application, the exchangeable As fraction (F1) decreased from approximately 4% in the control soil to nearly **0%**, whereas the weakly crystalline Fe–Al oxide-bound fraction (F3) increased to approximately **30–45%** during the incubation period, which indicated a transformation of As from more labile to relatively stable fractions. Compared with those in the CK group, the proportions of F3 in the SEP and nZVI treatment groups increased by 6.79–8.04% and 10.32–14.96%, respectively. After 120 days of culture, the S-nZVI treatment resulted in a more obvious morphological transformation, with the F1 proportion decreasing to 1.23% and the F3 proportion increasing to 21.56% and 22.30%, respectively. Under the 120% WHC condition, each passivation agent treatment also reduced the proportions of F1 and F2 and increased the proportions of stable forms such as F3 and F4. Among the group, S-nZVI-5 showed more stable morphological distribution characteristics throughout the entire culture process.

**Figure 5:**
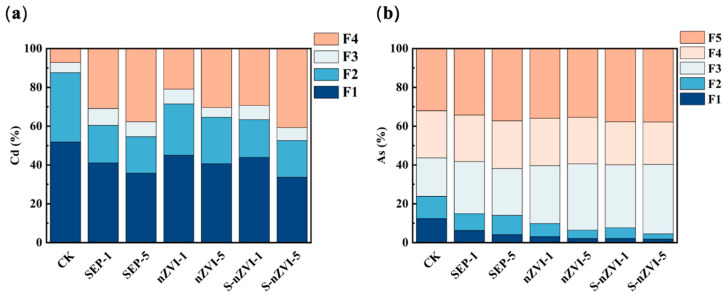
Effects of various treatments on the fractions of (**a**) Cd and (**b**) As in rhizosphere soil during the potted rice experiments.

The results of the morphological analysis of the rhizosphere soil in the potted rice experiment revealed that different passivant treatments altered the distributions of Cd and As in the rhizosphere soil (Figure 5). In terms of Cd, weakly acid-soluble Cd (F1) and reducible Cd (F2) were present in greater amounts in the rhizosphere soil of the CK group. Compared with the CK treatment, the SEP, nZVI, and S-nZVI treatments reduced the proportions of F1 and F2 and increased the proportion of residual Cd (F4). The proportion of F1 in the SEP treatment group decreased to 41.29–36.43%, and the proportion of F4 increased to 31.83–38.19%. S-nZVI treatment promoted the transformation of Cd into its stable form. Under the S-nZVI-5 treatment, the proportion of F4 increased to 41.43%, whereas the proportion of F1 decreased to 34.72%, which indicated more obvious morphological transformation characteristics. In terms of As, the proportions of nonspecifically adsorbed (F1) and specifically adsorbed (F2) As in the rhizosphere soil of the CK group were 11.84% and 12.19%, respectively, whereas the proportions of residual As (F5) and crystalline iron-aluminum oxide-bound As (F4) were lower. After the passivating agent was added, the proportions of F1 and F2 decreased in each treatment group, and the proportions of stable forms such as F3, F4, and F5 increased. Among the treatments, the nZVI and S-nZVI treatments had more significant effects on the distribution of As forms. Under the S-nZVI treatment, the proportion of residual As (F5) increased to 38.67%, which indicated that As was transformed into a stable form in the rhizosphere soil.

### 3.5. Effects on Rice Growth and Heavy Metal Accumulation

**Figure 6:**
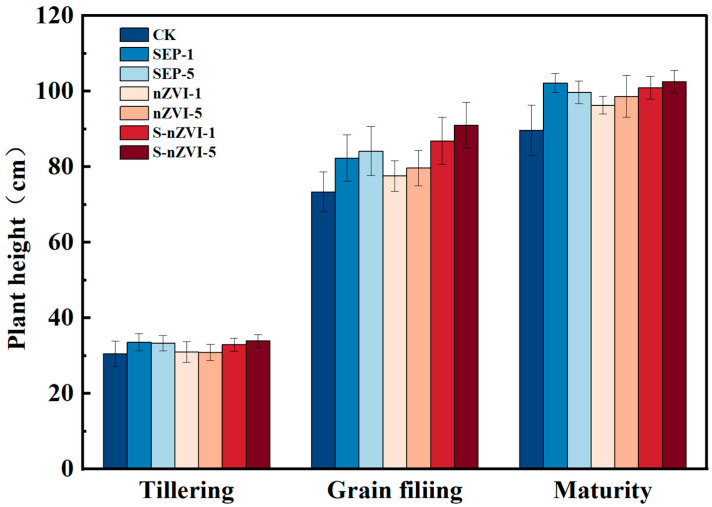
Effects of various treatments on the plant height of rice at various growth stages.

**Figure 7:**
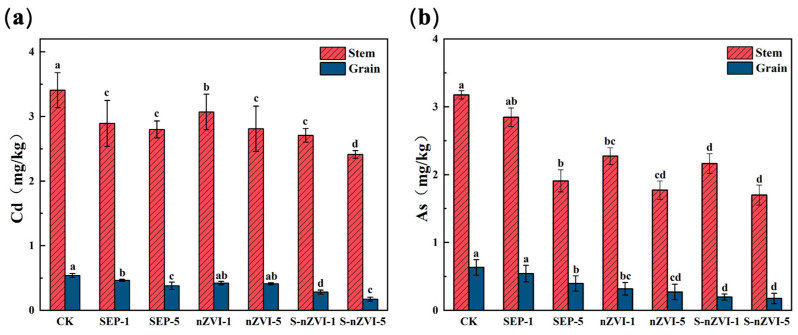
Effects of various treatments on As and Cd accumulation in rice stems and grains ((**a**): Cd content; (**b**): As content). (a, b, c, d indicate significant differences between groups at *p* < 0.05; ab indicates no significant difference from groups a and b, and so on.).

The results of the experiments on potted rice revealed that different passivant treatments significantly affected rice growth and the accumulation of Cd and As in the plants (Figure 6 and Figure 7, Table 2). During the tillering stage of the rice plants, the plant height of each treatment group was maintained at approximately 30 cm, and the difference between treatments was **not significant (*p* > 0.05)**. After the grain-filling period began, differences in plant height among the different treatments gradually emerged. Overall, the plant heights of the SEP and S-nZVI treatment groups were greater than those of the CK and nZVI treatment groups, and the plant height of the S-nZVI-5 treatment group reached 90.97 cm. At the maturity stage, the plant height of each treatment group further increased, and the trend difference remained consistent. The S-nZVI-5 treatment group had the greatest plant height, which was 102.46 cm.

Different passivant treatments reduced the Cd (Cd) and As (As) contents in rice stems and grains to varying extents. Compared with those in the CK group, the Cd concentrations in the rice stems decreased by **11.8–29.2%**, whereas the Cd concentrations in the rice grains decreased by **14.8–67.9%** across the different treatments **(*p* < 0.05)**. With respect to As, the concentrations in the stems decreased by **10.3–46.5%**, whereas those in the grains decreased by **14.3–72.2% (*p* < 0.05)**. Among all the treatment groups, the S-nZVI-5 group exhibited the strongest immobilization effect. In this treatment group, the Cd and As concentrations in the rice grains were **0.173 mg kg^−1^** and **0.176 mg kg^−1^**, respectively, which represented reductions of **67.9%** and **72.2% (*p* < 0.05)**, respectively, compared with those in the CK group. Enrichment coefficient analysis indicated that the enrichment coefficient of Cd in the rice stems was consistently greater than that in the grains under all the treatments, thus suggesting a preferential accumulation of Cd in vegetative tissues. In contrast, the application of S-nZVI effectively suppressed the enrichment of both Cd and As in the rice grains. Specifically, the enrichment coefficients of Cd and As in the grains under the S-nZVI-5 treatment were **0.558** and **0.058**, respectively, which represented the lowest values among all treatments.

## 4. Discussion

### 4.1. Material Structural Characteristics and Their Effects on Repair Performance

The results of this study show that sepiolite, as a carrier of nanoscale zero-valent iron, can significantly improve the structural stability and interfacial reaction performance of composite materials. SEM revealed that single nZVI particles are prone to agglomeration, whereas iron particles in the composite system are evenly dispersed on the surface of sepiolite, which indicates that the fibrous porous structure has a good spatial confinement effect on the nanoparticles. Partial iron oxides are formed on the surfaces of some nZVI particles because of oxidation and are partially aggregated into small clusters and supported on sepiolite, which is similar to the findings of previous studies [33]. Previous studies have generally reported that clay minerals or porous carriers can inhibit the aggregation of iron nanoparticles and delay oxidative deactivation through physical support and surface interactions [34,35]. Our findings reveal that nZVI nanoparticles are not only deposited on the surface but also distributed within the micropores of sepiolite. This unique intrapore loading not only increases the physical stability of the composite but also isolates the nZVI from rapid environmental oxidation, thereby preserving its reactivity.

The key parameters for evaluating the adsorption performance of a material include the specific surface area, pore structure size, and pore volume of the material. BET analysis revealed that the composite material has a high specific surface area and a developed mesoporous structure, which is generally considered to be beneficial for increasing the density of reaction sites on the surface of the material. The XRD results show that the composite process does not destroy the sepiolite crystal structure, but the Fe^0^ diffraction peak is weak, which reflects the high dispersion of iron particles. The typical diffraction peaks observed for SEP are similar to those in the present study [36,37,38]. These findings confirm that the synthesis process successfully reproduces the S-nZVI crystal properties that have been reported in the literature [39] and that the material preparation process is highly repeatable. XPS analysis confirmed the presence of multivalent iron species on the surface of the material, thus indicating that nanoscale zero-valent iron undergoes surface oxidation and forms iron (hydr) oxide during the reaction process. Previous studies have shown that this type of iron oxide has a strong adsorption capacity for As and can participate in the coprecipitation process of heavy metals [40,41]. Therefore, the composite material constructed in this study combines the carrier stabilization effect with iron-based reactivity to form an interface structure system with sustained reaction capabilities.

### 4.2. Proposed Immobilization Mechanisms of Cd and As in Soil by S-nZVI

The experimental results show that composite materials outperform single materials in the simultaneous immobilization of **As** and **Cd**. Studies have reported that clay mineral materials usually immobilize Cd^2+^ through surface adsorption and ion-exchange mechanisms [42], whereas iron-based materials play an important role in the **As** stabilization process [43]. The results of this study are consistent with these findings from the literature and also indicate that combining the two materials can improve the efficiency of simultaneous passivation. This synergistic effect may arise from the complementarity of the reaction pathways of the two materials. Sepiolite provides many negatively charged surface active sites, which are beneficial for the adsorption and exchange of Cd^2+^; nanoscale zero-valent iron promotes **As** adsorption and coprecipitation processes through redox reactions and the generation of iron oxides. Previous studies have shown that nanoiron materials are prone to agglomeration and rapid oxidation in soil, which limits their sustained reaction capabilities [44]. In this study, the composite system was revealed to significantly improve the dispersion of iron particles, which indicates that the carrier structure can delay its deactivation process, thereby improving the long-term repair effect of the material.

Soil pH and Eh are considered important environmental factors that control the migration and transformation of heavy metals. In this study, the application of S-nZVI led to an increase in soil pH and a decrease in Eh; these trends are consistent with previous observations for iron-based amendments. Although this general pattern aligns with existing knowledge, a distinct contribution of this work lies in the finding that S-nZVI more stably regulates pH and Eh over time, thus potentially offering more sustained and favorable conditions for the long-term immobilization of contaminants. Moreover, our results demonstrate that flooding conditions can increase the remediation efficiency of the composite by modulating the coupled pH–Eh dynamics, thus providing direct evidence that moisture regimes influence material performance in paddy soils. These insights not only deepen the understanding of the S-nZVI working mechanism under varying environmental conditions but also highlight its potential for stable, long-term application in complex agricultural systems.

The continuous extraction results show that the composite material can significantly reduce the proportion of more active exchangeable states in As and Cd and simultaneously promote their transformation into residual states and iron oxide combined states. Previous studies have generally reported that the conversion of heavy metals from active states to stable states is the key path for reducing bioavailability [45] and have shown that sepiolite can promote the transformation of Cd from the weak acid extraction state to the residual state [46]. Sepiolite in synthetic materials can electrostatically adsorb Cd^2+^ through negative surface charges. The iron oxide generated after oxidation of the loaded nanoscale zero-valent iron can fix Cd ions [47]. The results of this study are consistent with this theory and demonstrate that composite materials can promote this transformation process. In addition, the proportion of stable forms further increased under high-moisture conditions, thus indicating that moisture status may promote the stabilization of heavy metals by regulating the soil reducing environment; this finding is consistent with previous research that showed that adding 5% S-nZVI under 70% WHC conditions can reduce the available Cd in soil by 49.04% [48]. These results suggest that the repair effect of a composite material not only depends on the properties of the material but is also closely related to the soil moisture environment.

The rhizosphere environment is the core interface that controls heavy metal migration and plant uptake; thus, changes in the forms of As and Cd directly affect their bioavailability. Many studies have reported that exchangeable states and weakly adsorbed states usually represent metal pools that are more mobile and available to plants, whereas residual states and iron oxide-bound states are relatively stable [49,50]. Therefore, the conversion of heavy metals from active states to stable states is considered a key way to reduce ecological risks. In this study, S-nZVI significantly reduced the proportion of the active forms of As and Cd and promoted their transformation into stable forms, which indicated that the composite material effectively changed the geochemical fate of the metals in the soil. This result is attributed mainly to the iron (hydr)oxide generated during the corrosion process of nanoscale zero-valent iron, whose surface-rich hydroxyl sites can fix As through inner-layer complexation and coprecipitation [51,52]. With respect to Cd, the high specific surface area and cation-exchange sites provided by sepiolite promote rapid adsorption, whereas the participation of the iron phase improves the long-term stabilization effect. This combination of adsorption and iron-dominated reaction explains the superiority of composite systems over single materials. In addition, the decrease in the proportion of active forms in the rhizosphere is consistent with the decreases in As and Cd accumulation in rice, thus supporting the view that plant uptake is controlled by soil chemical forms [53]. Therefore, the repair effect of S-nZVI can be viewed as a stabilization process that progresses from adsorption to reduction to mineral binding rather than just short-term surface fixation. In summary, by controlling the dispersion and reaction path of nanoiron via the carrier, synergistic stabilization of As and Cd can be achieved, which provides a sustainable solution for the in situ remediation of multimetal complex contamination in agricultural environments.

### 4.3. Limitations and Future Prospects

In this study, a simultaneous remediation strategy for As and Cd composite pollution was developed from the perspective of material design. Previous research has focused mostly on the control of single-metal pollution; however, agricultural environments often exhibit multielement composite pollution. The results of this study show that composite materials can simultaneously reduce the effectiveness of As and Cd and promote their stable transformation, thus providing a new technical path for the remediation of composite-contaminated soil. At the agricultural application level, the results of the pot experiments indicate that composite materials not only reduce the accumulation of heavy metals in rice but also promote crop growth. Studies have shown that the alleviation of heavy metal stress usually helps restore plant physiological and metabolic functions [54]. This study revealed improved performance in the later stages of rice growth, which indicated that the composite material reduced the toxicity of heavy metals without negatively affecting the crop. Combined with the characteristics of the flooded rice field environment, the composite material showed good adaptability, which indicates its application potential for safe utilization in agriculture. Although this study revealed that composite materials have good repair effects, several limitations exist. First, this study was based mainly on laboratory culture and pot experiments, and long-term field verification was not conducted. Different environmental disturbances may affect the stability of the material. Second, different soil types vary greatly in terms of mineral composition and organic matter content, which may affect the universality of the remediation effect. In addition, the cost of material preparation and the feasibility of large-scale application still need to be evaluated. Although S-nZVI shows promise for soil remediation, the application of iron-based nanomaterials in agricultural ecosystems warrants the consideration of potential environmental risks, which may include (i) ecotoxicological effects on soil biota (e.g., earthworms and beneficial microorganisms) at high concentrations, (ii) potential mobilization of coexisting contaminants owing to changes in redox conditions, and (iii) effects on the long-term fate and transformation of nanoparticles in the soil environment. In future studies, the ecotoxicity of S-nZVI should be assessed, and its long-term effects on soil health and crop safety should be monitored before large-scale field application. The use of stabilizers or coatings to mitigate potential toxicity could also be explored. Future research can focus on long-term field trials to verify the stability of the restoration and explore the suitability of the materials for different soil types. Moreover, material modification and application method optimization should be combined to reduce application costs and improve repair efficiency. In addition, further analysis of the material interface reaction mechanism and its ecological effects will help promote the practical application of this type of material in agricultural pollution remediation.

## 5. Conclusions

In this study, a composite material, S-nZVI, that was loaded with nanoscale zero-valent iron was successfully prepared using sepiolite as a carrier, and its remediation performance for As- and Cd-composite-contaminated soil was systematically evaluated. The material characterization results indicate that sepiolite can effectively inhibit the agglomeration of nanoscale zero-valent iron particles and improve their dispersion stability while maintaining the integrity of the carrier crystal structure and significantly increasing the specific surface area and interfacial reactivity of the composite material. The results of soil culture experiments revealed that S-nZVI can significantly regulate soil pH and Eh conditions, reduce the availability of As and Cd in soil, and promote the conversion of heavy metals from active states to stable states. Mechanistic analysis revealed that S-nZVI constructs an “adsorption–reduction–coprecipitation” synergistic fixation mechanism by integrating the adsorption and ion exchange of sepiolite and the reduction and iron phase generation process of nanoscale zero-valent iron, thereby achieving simultaneous passivation of As and Cd. In potted rice experiments, S-nZVI not only effectively reduced the bioavailability of As and Cd in rhizosphere soil but also significantly inhibited their accumulation in rice stems and grains and promoted rice growth. These results show that the composite material has good environmental adaptability and risk control capabilities in crop planting systems. Overall, the S-nZVI composite material constructed in this study provides a new technical approach with a synergistic fixation effect for the remediation of As and Cd complex-contaminated agricultural soil, which is highly important for ensuring agricultural ecological security and food security.

## Figures and Tables

**Table 1:** Basic properties and heavy metal contents of the tested soil.

	pH	SOC	Total Content (mg/kg)		Clay (%)	Silt (%)	Total Content (g/kg)		
			As	Cd			N	P	K
Tested soil	5.65	11.51	132.31	3.2	12.8	75.45	0.82	0.76	13.49

**Table 2:** Enrichment coefficients of As and Cd in stems and grains.

Treatment	Stem Cd Enrichment Coefficient	Grain Cd Enrichment Coefficient	Stem As Enrichment Coefficient	Grain Cd Enrichment Coefficient
CK	5.245	0.829	0.278	0.055
SEP-1	5.579	0.895	0.399	0.076
SEP-5	6.850	0.928	0.321	0.067
nZVI-1	5.933	0.814	0.431	0.060
nZVI-5	7.267	1.062	0.463	0.071
S-nZVI-1	7.123	0.739	0.432	0.039
S-nZVI-5	7.792	0.558	0.556	0.058

## Data Availability

The original contributions presented in this study are included in the article/Supplementary Materials. Further inquiries can be directed to the corresponding author.

## Supplementary Materials

The following supporting information can be downloaded at: https://www.mdpi.com/article/10.3390/toxics14040301/s1, Figure S1: Scanning electron microscope (SEM) image: (a) SEP; (b) S-nZVI. Energy Dispersive X-Ray Spectroscopy (EDX) image: (c) S-nZVI; Figure S2: title; Figure S3: XRD patterns of SEP, nZVI, and S-nZVI; Figure S4: XPS spectra of S-nZVI: (a) O 1s; (b) Fe 2p; (c) C 1s; Figure S5: Dynamic changes of soil pH and Eh under 50% WHC and 120% WHC treatments; Figure S6: Effects on Cd speciation in soil at 50% WHC during soil incubation experimental over different days; Figure S7: Effects on Cd speciation in soil at 120% WHC during soil incubation experimental over different days; Figure S8: Effects on As speciation in soil at 50% WHC during soil incubation experimental over different days; Figure S9: Effects on As speciation in soil at 120% WHC during soil incubation experimental over different days; Table S1: Advantages and limitations of different remediation materials or approaches; Table S2: Experimental Design for Soil Incubation.
