# Supplementary material for: Synchronous Immobilization of Arsenic and Cadmium in Agricultural Soils by Sepiolite-Supported Nanoscale Zero-Valent Iron
Source: Toxics. 2026 Mar 31;14(4):301. doi: 10.3390/toxics14040301 (PMC13120508; doi:10.3390/toxics14040301)
Supplement: Supplementary file 1 [file toxics-14-00301-s001.zip › toxics-4199789-supplementary.pdf]

Supplementary Materials for

Synchronous immobilization of arsenic and cadmium in agri-cultural soils by sepiolite-supported nanoscale zero-valent iron

Kuiru Li[1, †], Tieguang He[2, †], Yan Wang [3], Xinru Wang[1], Donghuan Lei[1], Lijuan Li[1, *]

1 College of Agriculture, Guangxi University/Guangxi Key Laboratory of Agroenvironment and Agroproduct Safety, Nanning 530004, China; yllkr1663229302@163.com(K.L.); 1477564847@qq.com(X.W.); 2033085103@qq.com(D.L.).

2 Agricultural Resources and Environmental Research Institute, Guangxi Academy of Agricultural Scienc-es/Guangxi Key Laboratory of Arable Land Conservation, Nanning 530007, China; tghe118@163.com (T.H.).

3 College of Environmental Science and Engineering, China West Normal University, Nanchong 637009, China; 1348551719@qq.com (Y.W.).

*Correspondence: Lilijuan@gxu.edu.cn(L.L.); Tel.: +86 178 1201 3979

These authors contributed equally to this work

This file includes:

Figs. S1 to S9

Tables S1 to S2

Table S1: Advantages and limitations of different remediation materials or approaches

Material / Approach	Advantages	Limitations	References
Clay Minerals	Strong ion exchange and physical adsorption capabilities for cadmium (Cd), resulting in good fixation.	Limited fixation effect on arsenic (As), which exists in an anionic form.	[11-13]
Iron-Based Materials	Effectively regulates the valence state of arsenic and promotes its fixation.	The long-term stabilizing effect on cadmium may weaken due to oxidative deactivation or changes in environmental conditions.	[14-16]
Hydroxyapatite	High fixation efficiency for cadmium, reaching up to 85%.	Promotes the activation and release of arsenic.	[17]
Lime (pH Adjustment)	Promotes cadmium precipitation by adjusting the pH.	Enhances the dissolution and migration ability of arsenic.	[17]
Flooded/Reducing Conditions	Creates an environment favorable for the fixation of cadmium.	Triggers the conversion of less toxic As(V) into highly toxic As(III).	[18]
Plant Uptake Mechanisms	(Describes the natural interaction between the two elements)	Arsenic promotes plant absorption of cadmium; cadmium inhibits arsenic transport, leading to a significant increase in combined toxicity.	[19]

Table S2: Experimental Design for Soil Incubation

Group	Material	Application Rate	Water Regime	Replicates
CK	No passivator	0 g/kg	50% WHC / 120% WHC	3
SEP-1	Sepiolite	1 g/kg (0.1%)	50% WHC / 120% WHC	3
SEP-5	Sepiolite	5 g/kg (0.5%)	50% WHC / 120% WHC	3
nZVI-1	Nano-zero-valent iron	1 g/kg (0.1%)	50% WHC / 120% WHC	3
nZVI-5	Nano-zero-valent iron	5 g/kg (0.5%)	50% WHC / 120% WHC	3
S-nZVI-1	Modified S-nZVI	1 g/kg (0.1%)	50% WHC / 120% WHC	3
S-nZVI-5	Modified S-nZVI	5 g/kg (0.5%)	50% WHC / 120% WHC	3

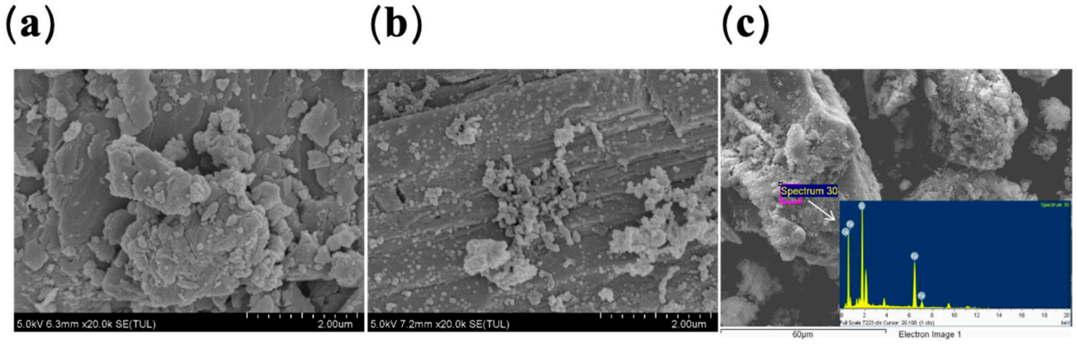

Figure S1: Scanning electron microscope (SEM) image: (a) SEP; (b) S-nZVI. Energy Dispersive X-Ray Spectroscopy(EDX) image: (c) S-nZVI.

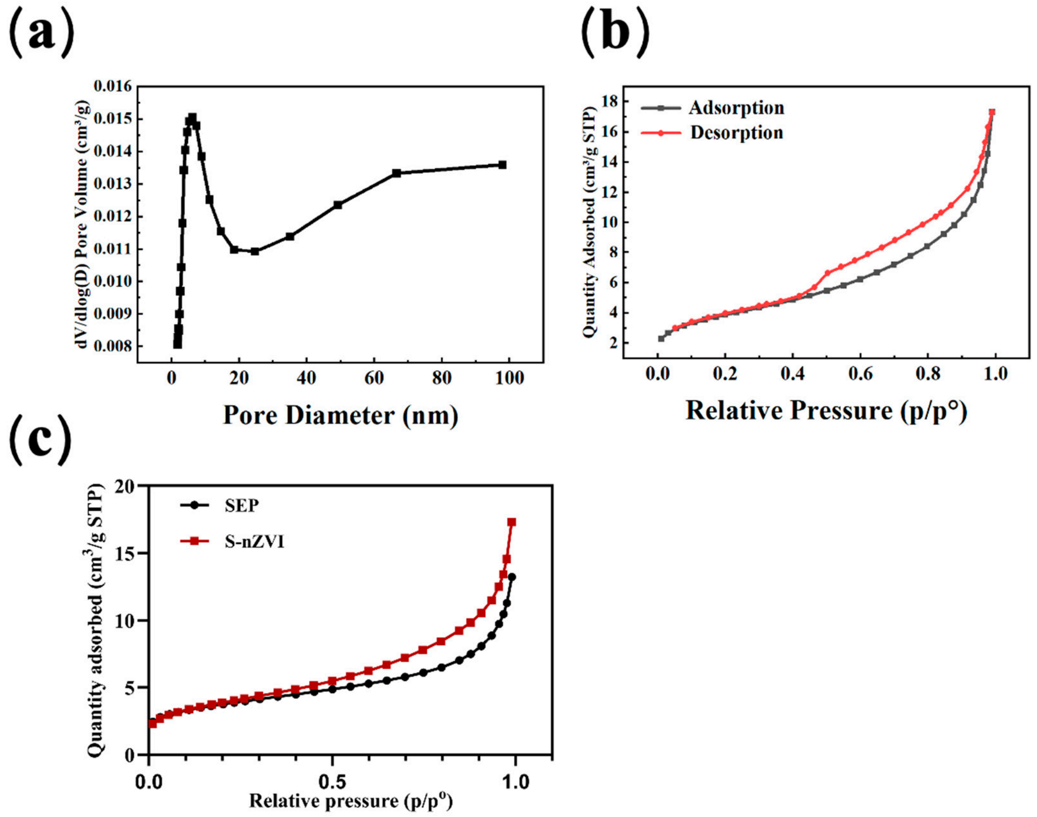

Figure S2: Textural characterization of freshly prepared S-nZVI: (a) pore size distribution, (b) N_2 adsorption–desorption isotherms. (c) N_2 adsorption–desorption isotherms of SEP and S-nZVI.

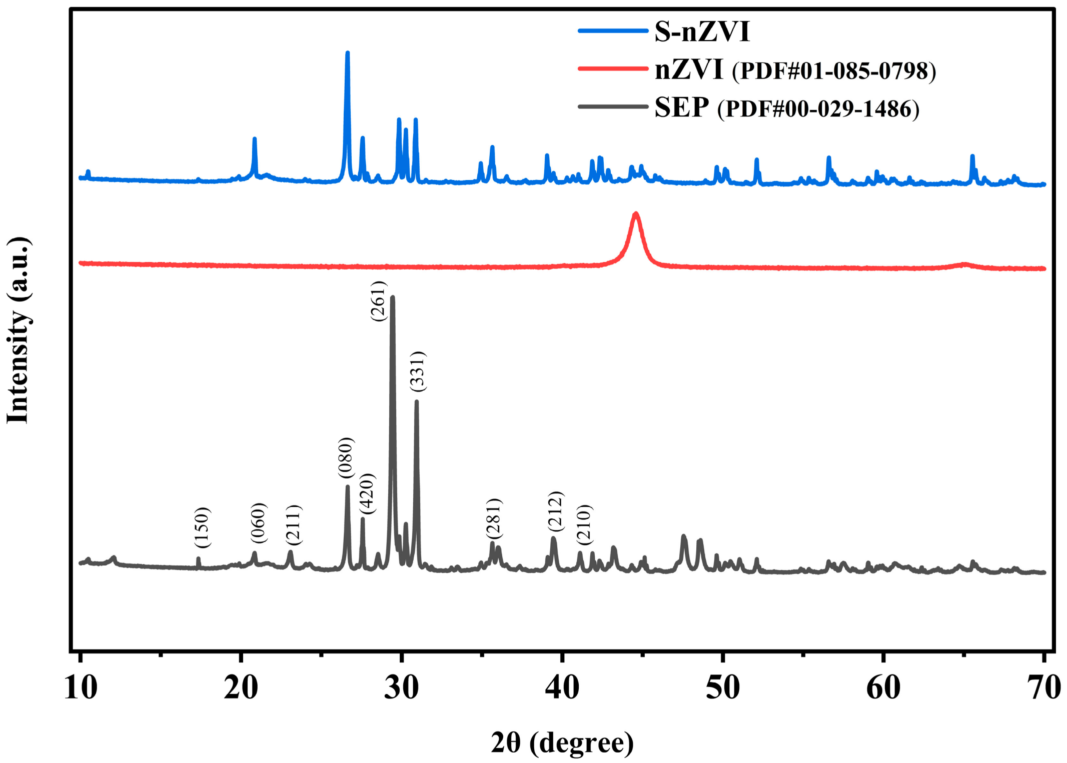

Figure S3:XRD patterns of SEP, nZVI, and S-nZVI.

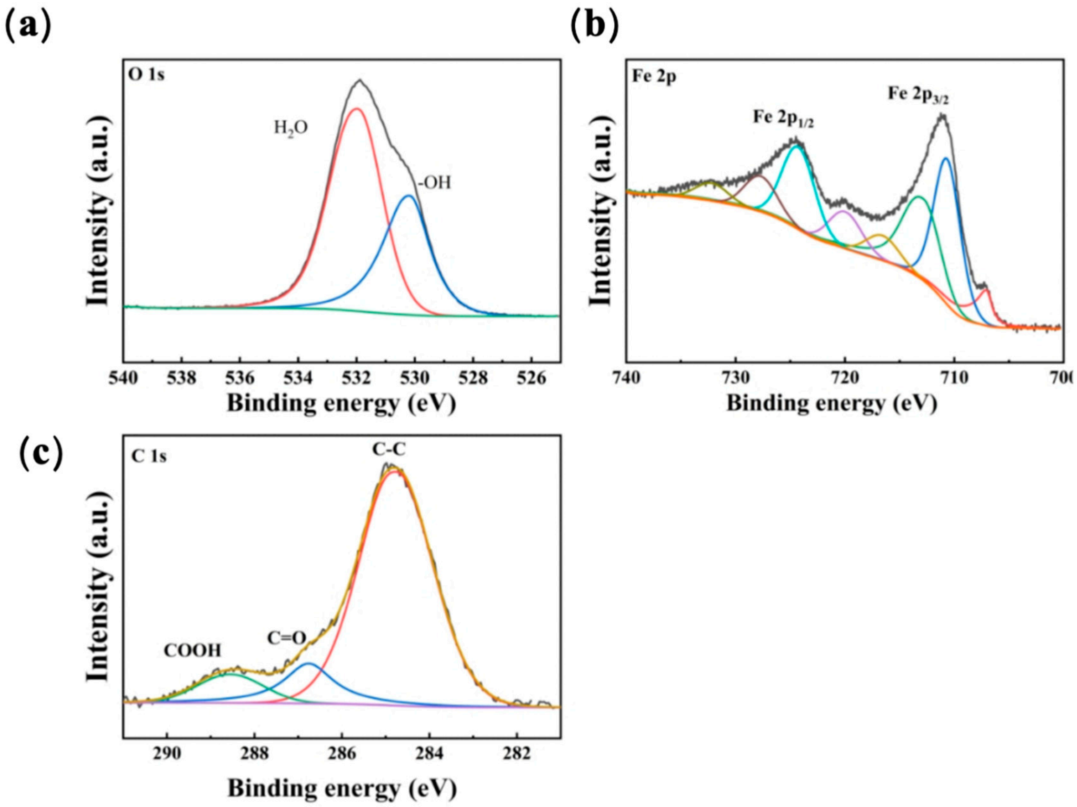

Figure S4:XPS spectra of S-nZVI: (a)O 1s; (b)Fe 2p;(c)C 1s.

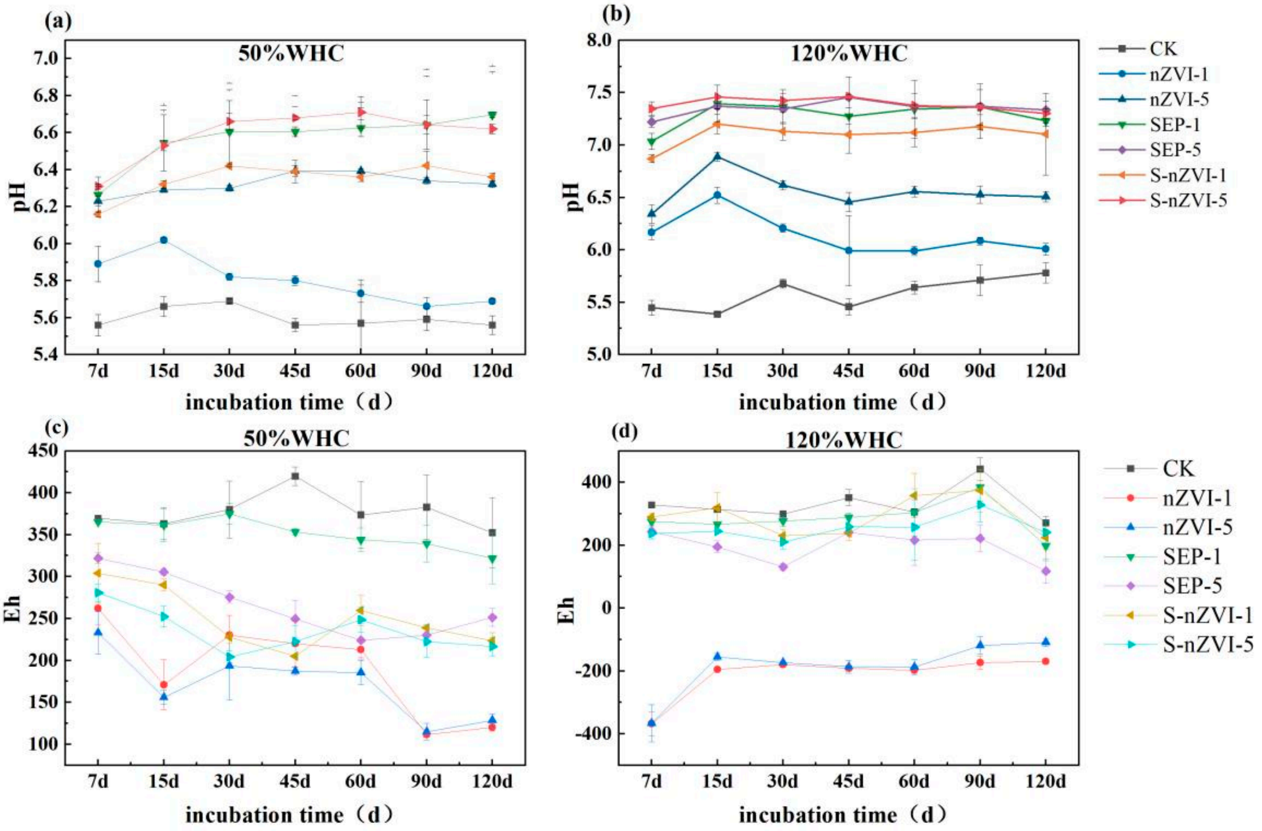

Figure S5: Dynamic changes of soil pH and Eh under 50% WHC and 120% WHC treatments

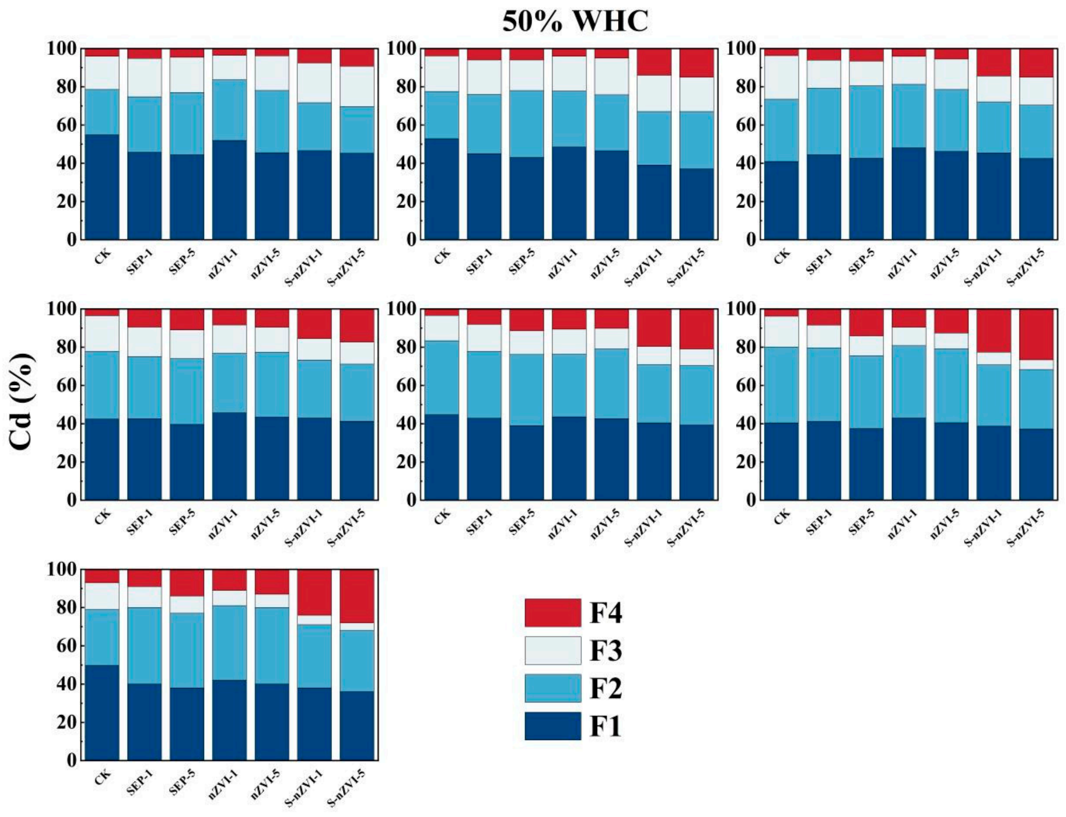

Figure S6: Effects on Cd speciation in soil at 50% WHC during soil incubation experimental over different days.

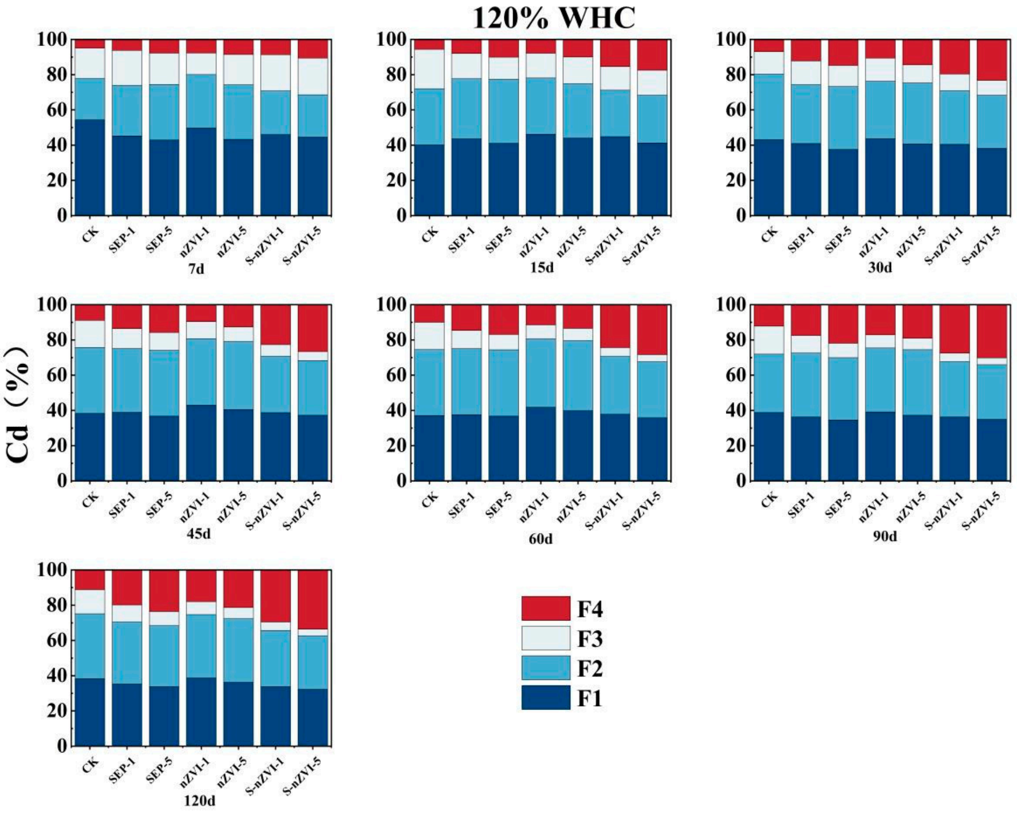

Figure S7: Effects on Cd speciation in soil at 120% WHC during soil incubation experimental over different days.

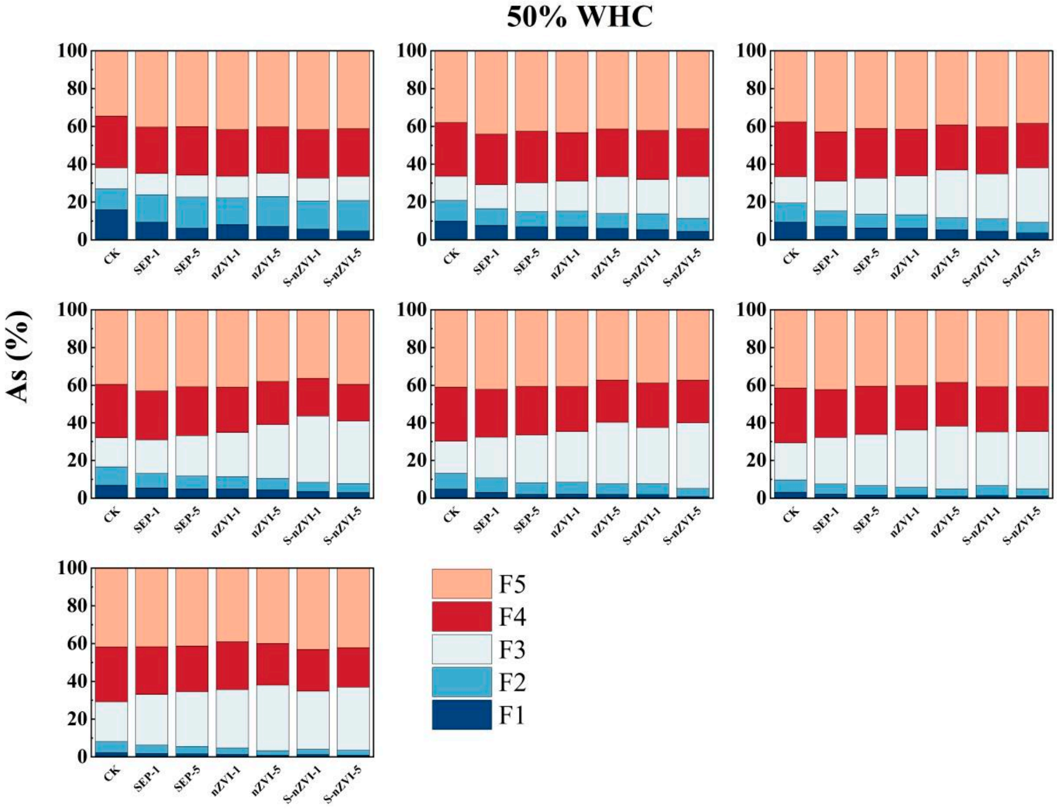

Figure S8: Effects on As speciation in soil at 50% WHC during soil incubation experimental over different days.

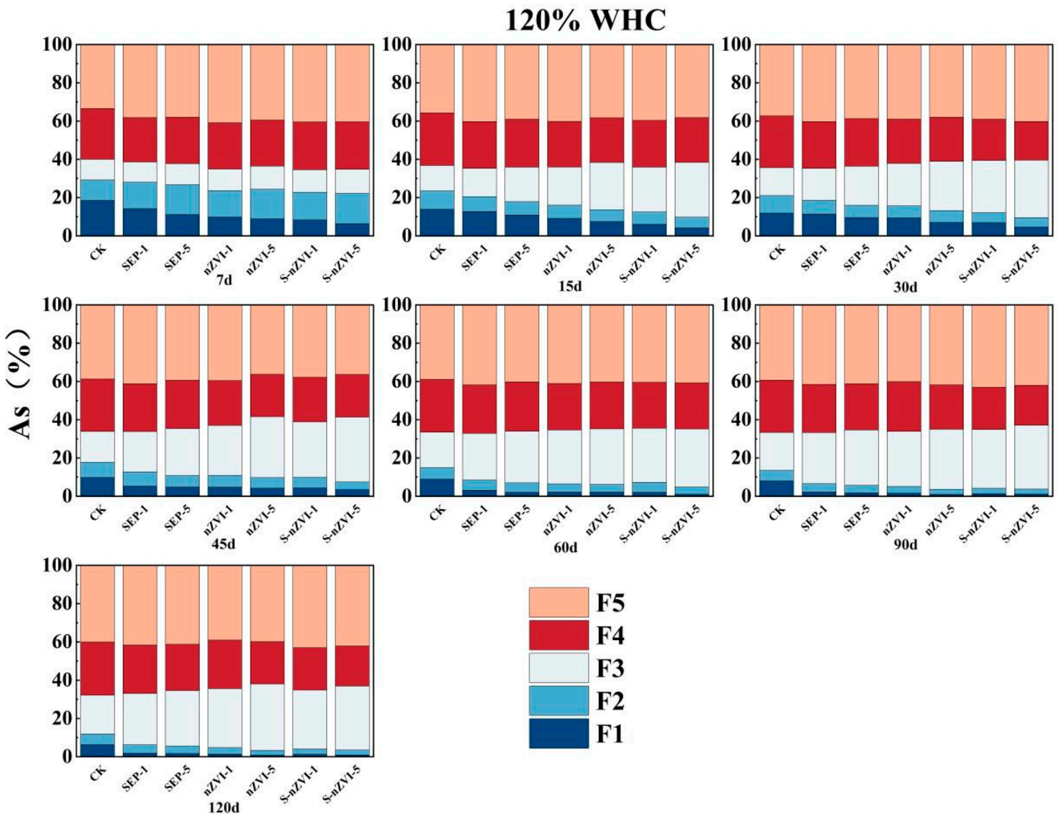

Figure S9: Effects on As speciation in soil at 120% WHC during soil incubation experimental over different days.
